# Supplementary material for: The non-catalytic DNA polymerase ε subunit is an NPF motif recognition protein
Source: Nat Commun. 2025 Dec 13;17:586. doi: 10.1038/s41467-025-67284-5 (PMC12808100; doi:10.1038/s41467-025-67284-5)
Supplement: Supplementary file 1 — Supplementary Information [file 41467_2025_67284_MOESM1_ESM.pdf]

Supplementary Information

The non-catalytic DNA polymerase ε subunit is an NPF motif recognition protein

Salla Keskitalo[1 *], Boglarka Zambo[2, 3 *], Dicle Malaymar Pinar[1], Antti Tuhkala[1], Kari Salokas[1], Tanja Turunen[1], Norbert Deutsch[4], Norman Davey[5], Zsuzsanna Dosztányi[4], Markku Varjosalo[1], Gergo Gogl[2, 3]

*authors contributed equally

Corresponding authors: Markku Varjosalo: markku.varjosalo@helsinki.fi, Gergo Gogl: gergo.gogl@univ-cotedazur.fr

[1] Institute of Biotechnology, Helsinki Institute of Life Science HiLIFE, University of Helsinki, Helsinki, Finland
[2] Institut de Génétique et de Biologie Moléculaire et Cellulaire (IGBMC), INSERM U1258/CNRS UMR 7104/Université de Strasbourg, Illkirch, France
[3] Institut de Biologie Valrose – iBV, Université Côte d'Azur, CNRS UMR7277, Inserm U1091, Nice, France
[4] Department of Biochemistry, ELTE Eötvös Loránd University, Pázmány Péter Sétány 1/c, H-1117 Budapest, Hungary
[5] Institute of Cancer Research, Chester Beatty Laboratories, 237 Fulham Rd, Chelsea, London SW3 6JB, UK

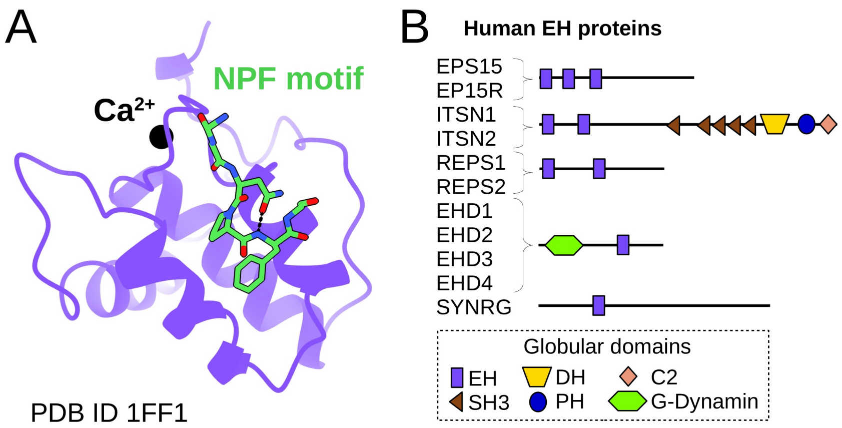

Supplementary Figure 1, EH proteins and NPF motif binding. (A) Structural snapshot of an interaction formed between an EH domain and an NPF motif. (B) Domain architecture of the human EH protein family, based on UniProtKB annotations.

A

B

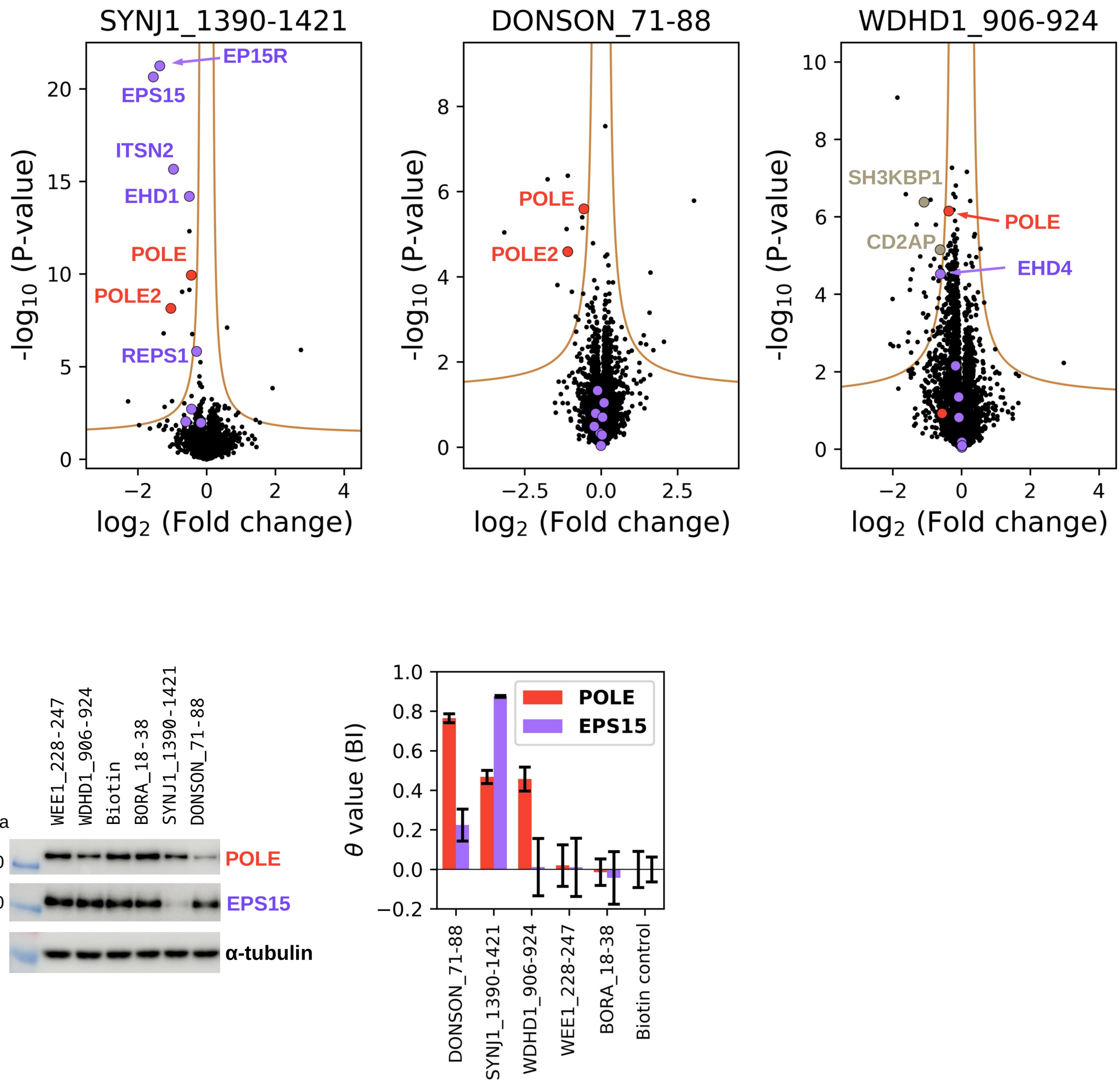

Supplementary Figure 2, Complementary nHU experiments confirm the interaction between multiple NPF motifs and POLE/POLE2. (A) Independent nHU-MS measurements carried out with three NPF motif peptide baits. Experiments were carried out with 2 biological and 3 technical replicates (total N = 6). (B) Summary of validation experiments analyzed by Western blot. The binding of endogenous POLE and EPS15 is monitored for five selected NPF motifs at a fixed bait concentration. The binding of POLE is clearly detected in the case of bait peptides taken from DONSON, WDHD1, and SYNJ1, while EPS15 could only interact with SYNJ1 and to a lesser degree DONSON. Mean and standard deviation θ values are shown based on Western blots technical duplicates (N=2). Source data can be found in Supplementary Figure 3, Supplementary Data 1, 2, and in the Source Data file.

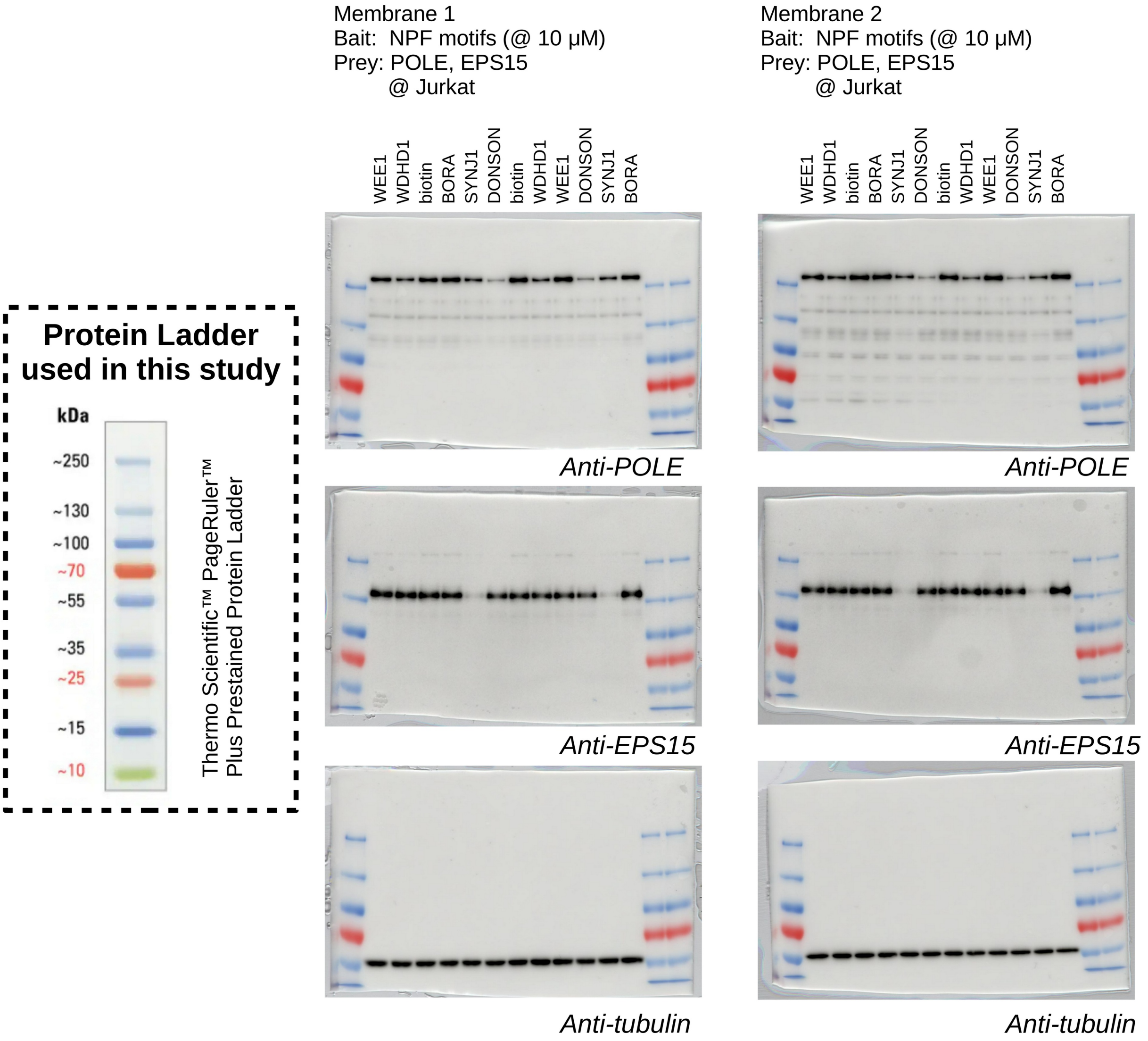

Supplementary Figure 3, Raw data of the nHU-WB experiments shown in Supplementary Figure 2. Two independent experiments are loaded on each blot and Western blots were performed in technical duplicates. Western blot images are shown as overlay of luminescence and colorimetric photos. The same protein marker is used in the entire study for which the manufacturer's reference image is included on the left side. Source data can be found in the Source Data file.

Measured θ w/ SYNJ1_1390-1421			
**prey**	**MS exp1**	**MS exp2**	**WB**
POLE2	0.58	0.51	N/D
POLE	0.36	0.27	0.47
EPS15	0.70	0.66	0.88
EP15R	0.78	0.61	N/D
EHD1	0.41	0.29	N/D
ITSN2	0.57	0.49	N/D
MIEF1	0.72	0.58	N/D
PCMT1	0.52	0.39	N/D
PPP2R2A	0.23	0.25	N/D

Measured θ w/ DONSON_71-88			
**prey**	**MS exp1**	**MS exp2**	**WB**
POLE2	0.56	0.53	N/D
POLE	0.50	0.32	0.76
SH3KBP1	0.63	0.53	N/D
CD2AP	0.58	0.35	N/D

Measured θ w/ WDHD1_906-924			
**prey**	**MS exp1**	**MS exp2**	**WB**
POLE2	0.47	0.32	N/D
POLE	0.29	0.22	0.46

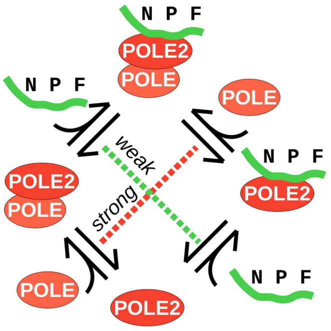

POLE2-NPF interaction

$C_{\text{NPF bait}} \approx 10\ \mu M$

$K_{\text{app, NPF-POLE2}} < 1 - 20\ \mu\text{M}$

POLE-POLE2 interaction

$C_{\text{POLE}} \approx C_{\text{POLE2}} \approx 1$ nM

$\theta_{\text{POLE2}} > \theta_{\text{POLE}}$ for all NPF motifs

$\Delta\theta_{\text{POLE2-POLE}} = 0.23\text{-}0.14$

$K_{\text{app, POLE-POLE2}} = 0.3\text{-}0.1$ nM

$$K_{\text{app, POLE-POLE2}} = \frac{(C_{\text{POLE2}} * \theta_{\text{POLE2}} - C_{\text{POLE}} * \theta_{\text{POLE}}) * (\theta_{\text{POLE2}} - \theta_{\text{POLE}})}{\theta_{\text{POLE2}} * \theta_{\text{POLE}}}$$

Supplementary Figure 4, Based on apparent affinities, POLE2 is suspected to bind to NPF motifs directly. The measured degree of binding is shown for all partners that both independent experiment identified to bind to their cognate partner. In all MS experiments, the fractional depletion of POLE2 is higher than of POLE. This is consistent with a model according to which POLE2 directly binds to various NPF motifs with affinities in the ~ µM regime, while it also binds to POLE with a dissociation constant in the ~ nM regime.

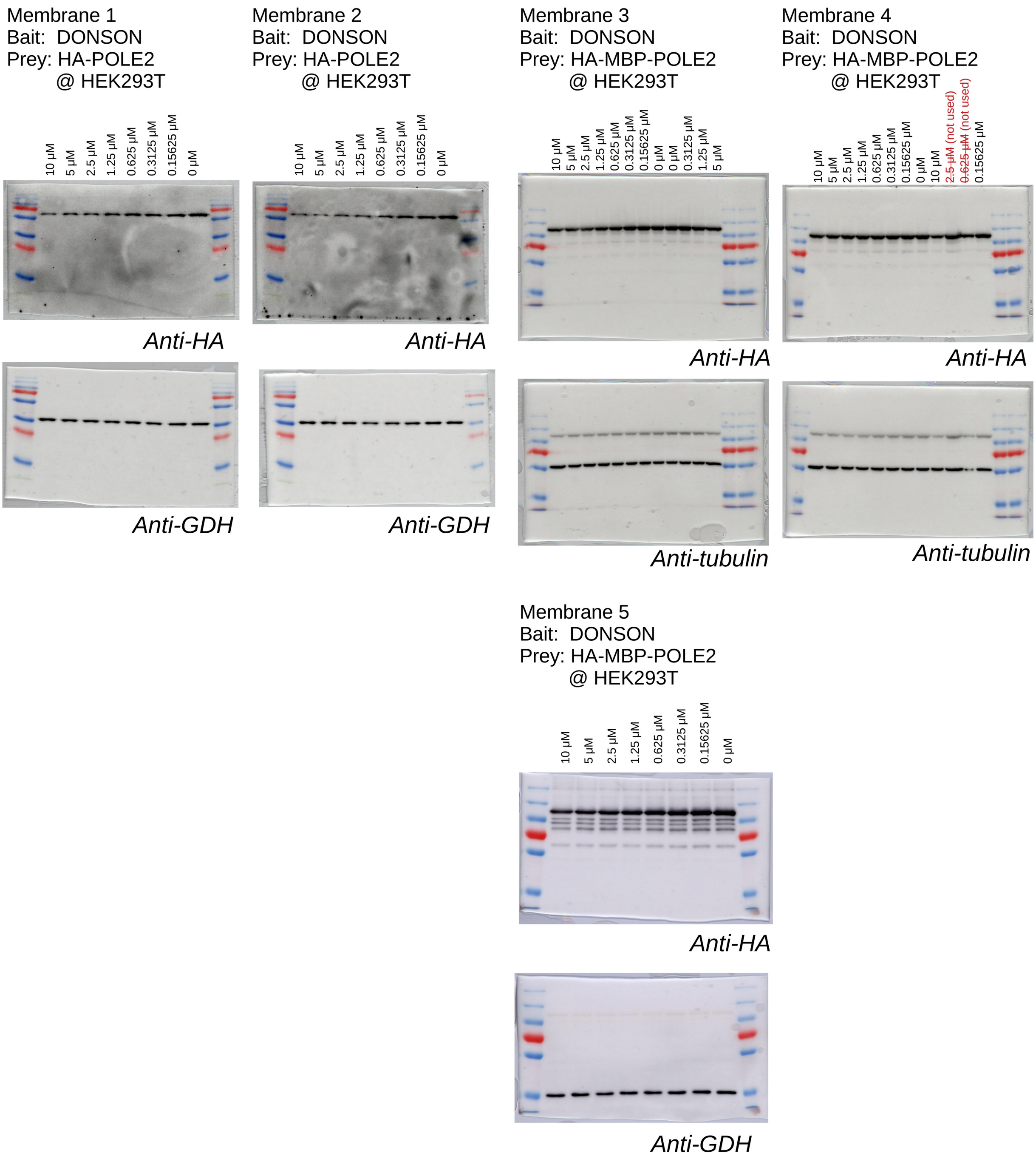

Supplementary Figure 5, Raw data of the nHU-WB experiments shown in Figure 1. Western blots were performed in technical duplicates or triplicates. Western blot images are shown as overlay of luminescence and colorimetric photos. Source data can be found in the Source Data file.

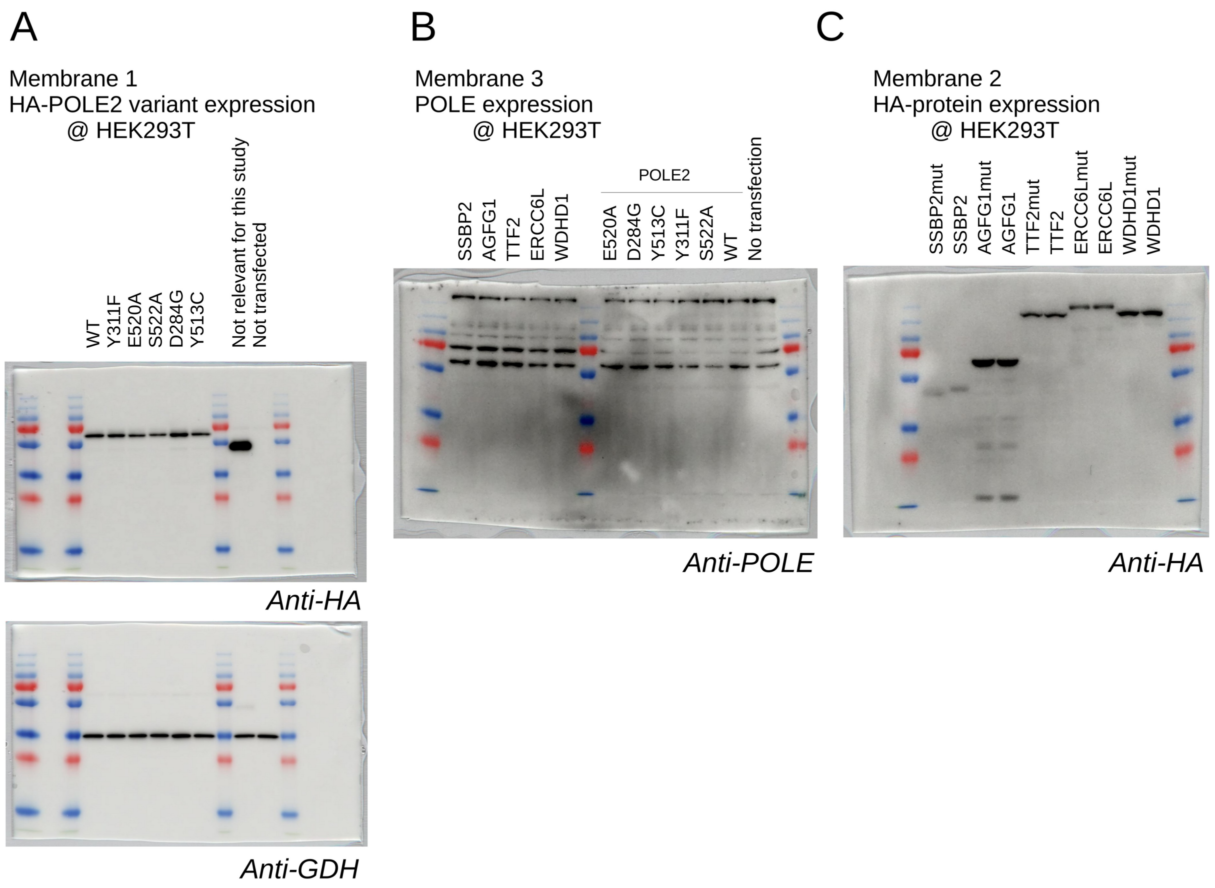

Supplementary Figure 6, Expression analysis of transfected proteins transiently expressed in HEK293T cells. (A) Neither the expression level of POLE2 (A), POLE (B), nor its partners (C) changed substantially upon the introduction of mutations in POLE2, or NPF motifs. Western blot images are shown as overlay of luminescence and colorimetric photos. Source data can be found in the Source Data file.

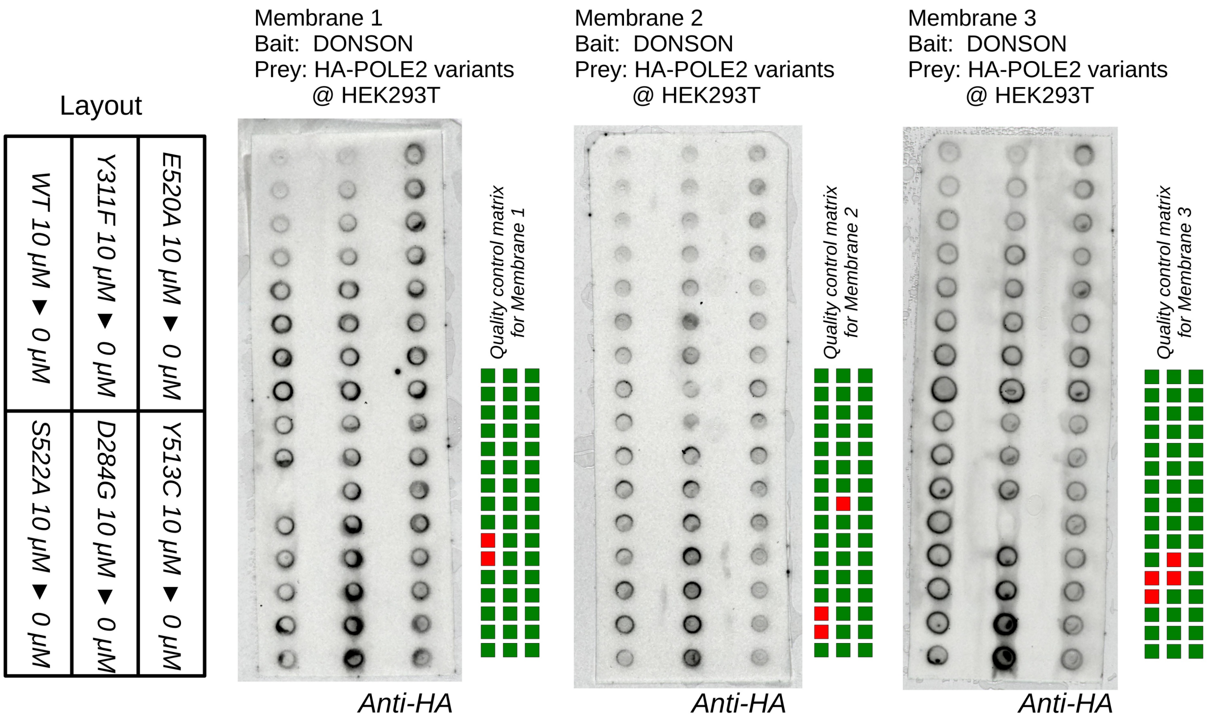

Supplementary Figure 7, Binding of DONSON to POLE2 mutants, measured with nHU experiments coupled with dot blot analysis. On a 48 dot screen, 8-point nHU titration experiments of six POLE2 variant is included. Dot blots were performed in technical triplicates. Due to technical difficulties, such as complete or partial signal loss due to puncture of membrane under vacuum, specific wells were omitted from analysis. This is indicated by a quality control matrix on the right side of each membrane. Source data can be found in the Source Data file.

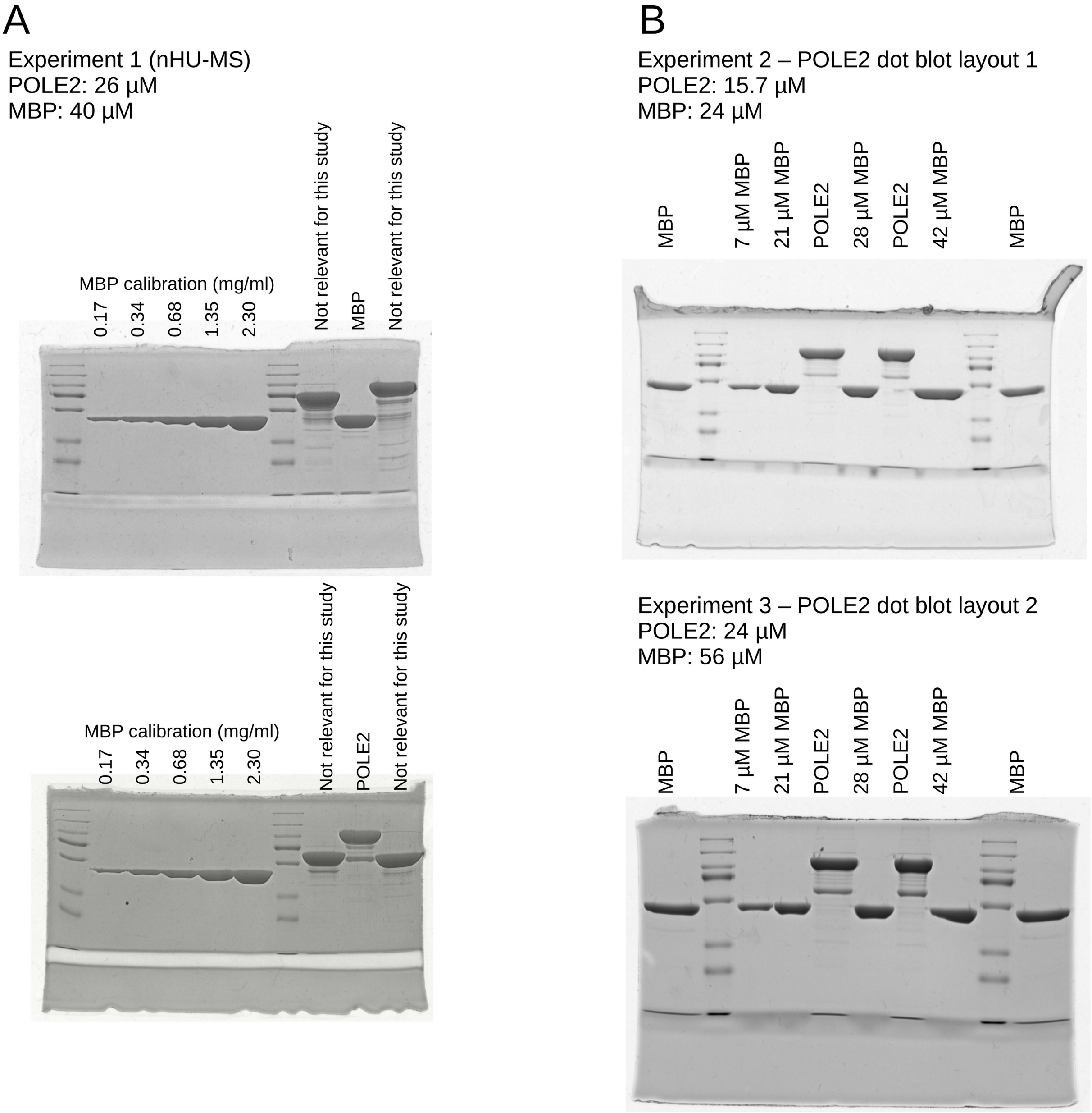

Supplementary Figure 8, Bait concentration measurement of nHU experiments carried out with protein baits. Bait concentrations of independent nHU experiments analyzed by MS (A), or dot blot (B) were analyzed independently. On each Coomassie-stained gel, a series of known amounts of purified MBP protein is loaded as a calibration standard alongside the eluted protein samples. Based on densitometry and the known molecular weight difference between MBP and the proteins of interest, the bait concentration can be deduced. Source data can be found in the Source Data file.

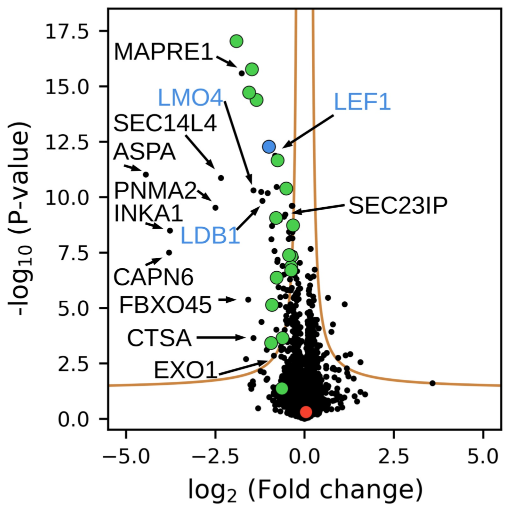

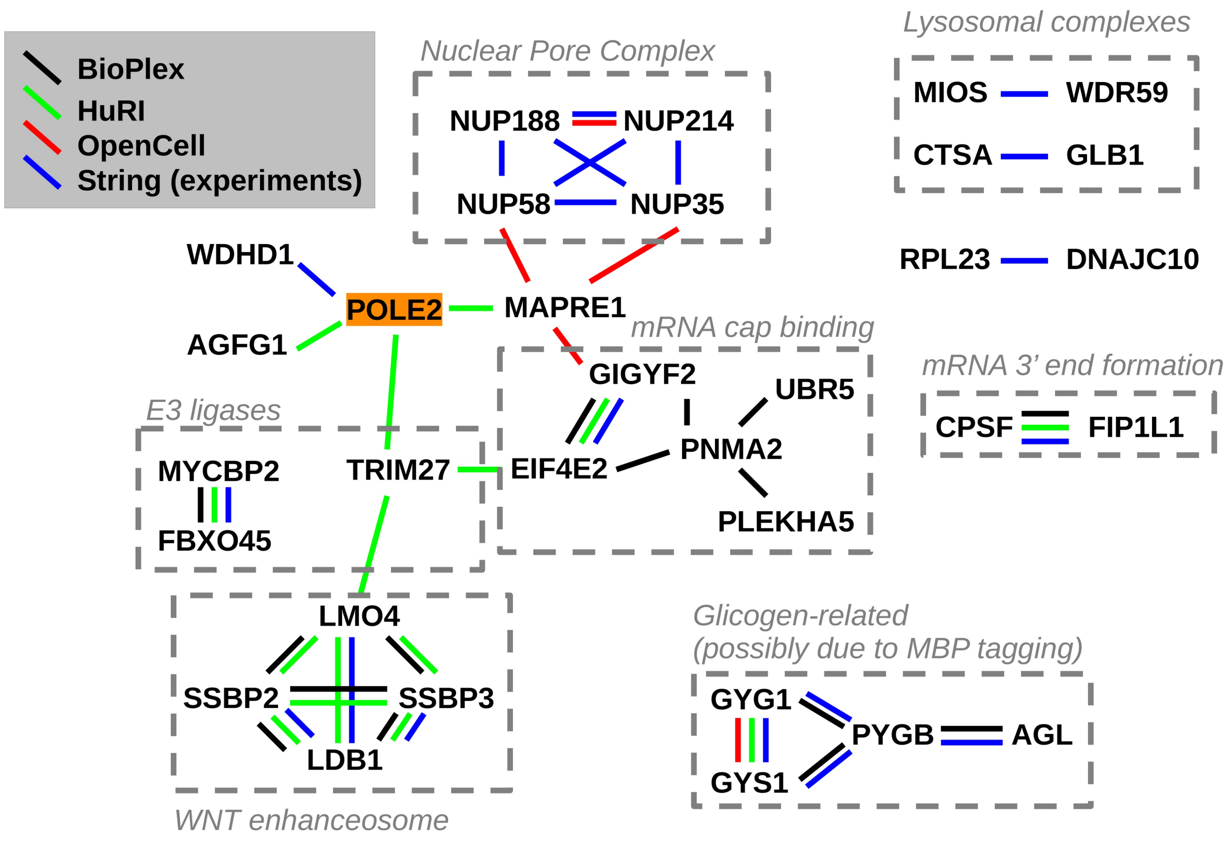

Supplementary Figure 9, Additional details for the proteome-wide interaction network of POLE2. (top) Other notable partners identified to bind to POLE2 that could not be showed in Figure 3 for clarity. Partners marked in blue are part of the WNT-enhanceosome. Coloring is exactly as shown on Figure 3. (bottom) Identified complexes among POLE2 partners. Source data can be found in Supplementary Data 1, and in the Source Data file.

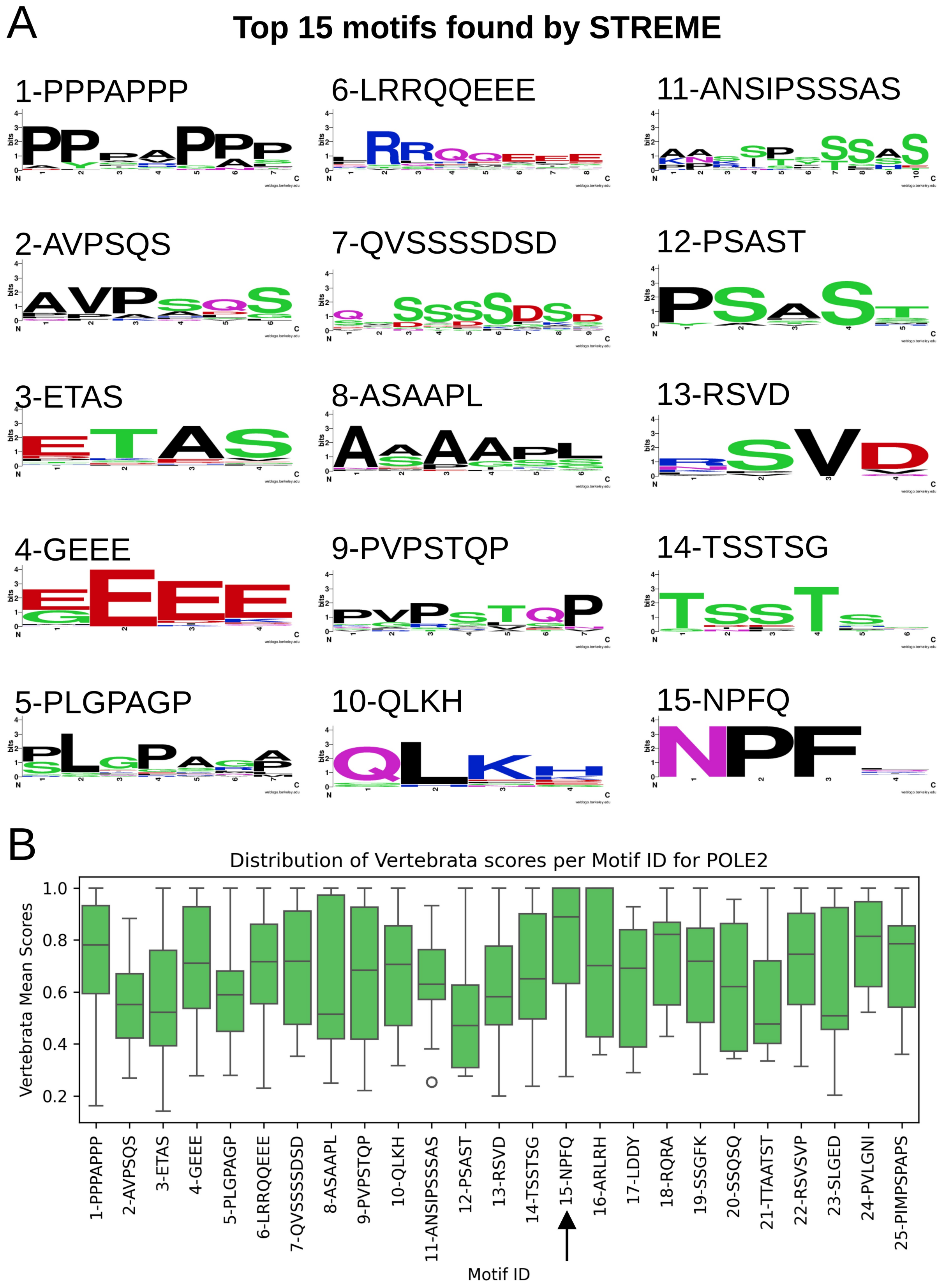

Supplementary Figure 10, Enriched motifs in the sequences of POLE2 partners. (A) The consensus logo of the top 15 motif classes identified by STREME using the disordered sequences of the partner proteins. (B) Taking into consideration the average motif evolutionary conservation within each identified motif class, the most conserved motif class was identified as the NPFQ motif, originally ranked as #15 most enriched motif type. Source data can be found in Supplementary Data 3, and in the Source Data file.

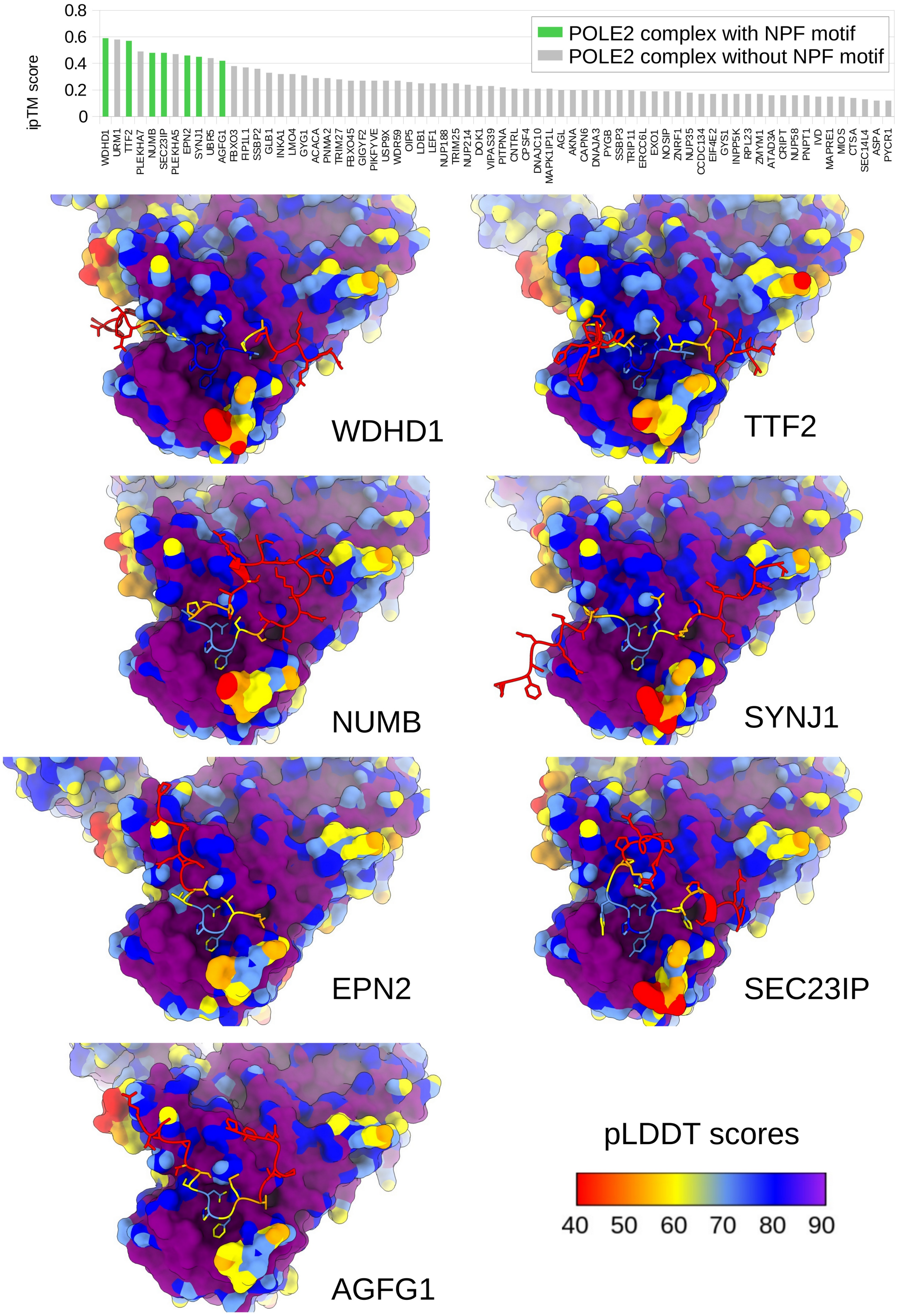

Supplementary Figure 11, NPF motifs are predicted to bind to POLE2 with high confidence. (top) Interface scores (ipTM) of binary AF3 predictions show that partners with identified NPF motifs scored among the best out of all POLE2 partners. (bottom) Only the predicted NPF/NPY motifs are shown in these illustrations out of the binary AF3 predictions between pairs of full-length proteins. The surface of POLE2, as well as the predicted motifs of partner proteins are colored according to their pLDDT scores. High confidence is indicated by much higher local pLDDT scores at the core NPF/NPY motifs than of their surrounding flanking sequences. Source data can be found in the Source Data file.

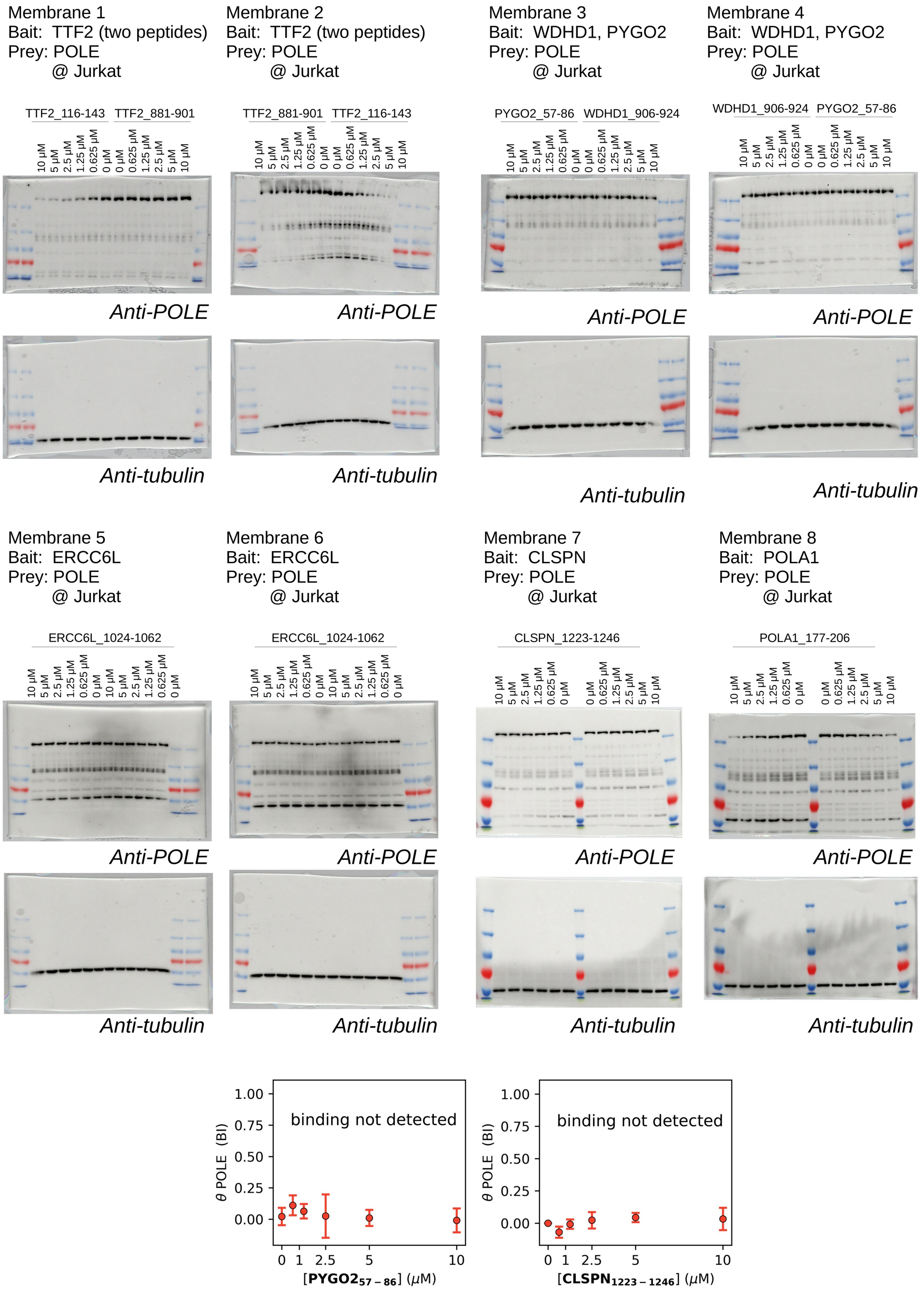

Supplementary Figure 12, NPF motifs taken out of selected identified full-length partners bind to POLE. (top) Titration nHU experiments with various NPF peptide baits were analyzed by Western blot to measure POLE depletion. Western blots were performed in technical duplicates. In the case of ERCC6L, the experiment was carried out in duplicates. Western blot images are shown as overlay of luminescence and colorimetric photos. (bottom) The NPF motifs of PYGO2 and CLSPN were found to be unable to bind to endogenous POLE. This is consistent with the finding of the MS results shown in Figure 1, as well as in Figure 3. Source data can be found in Supplementary Data 2, and in the Source Data file.

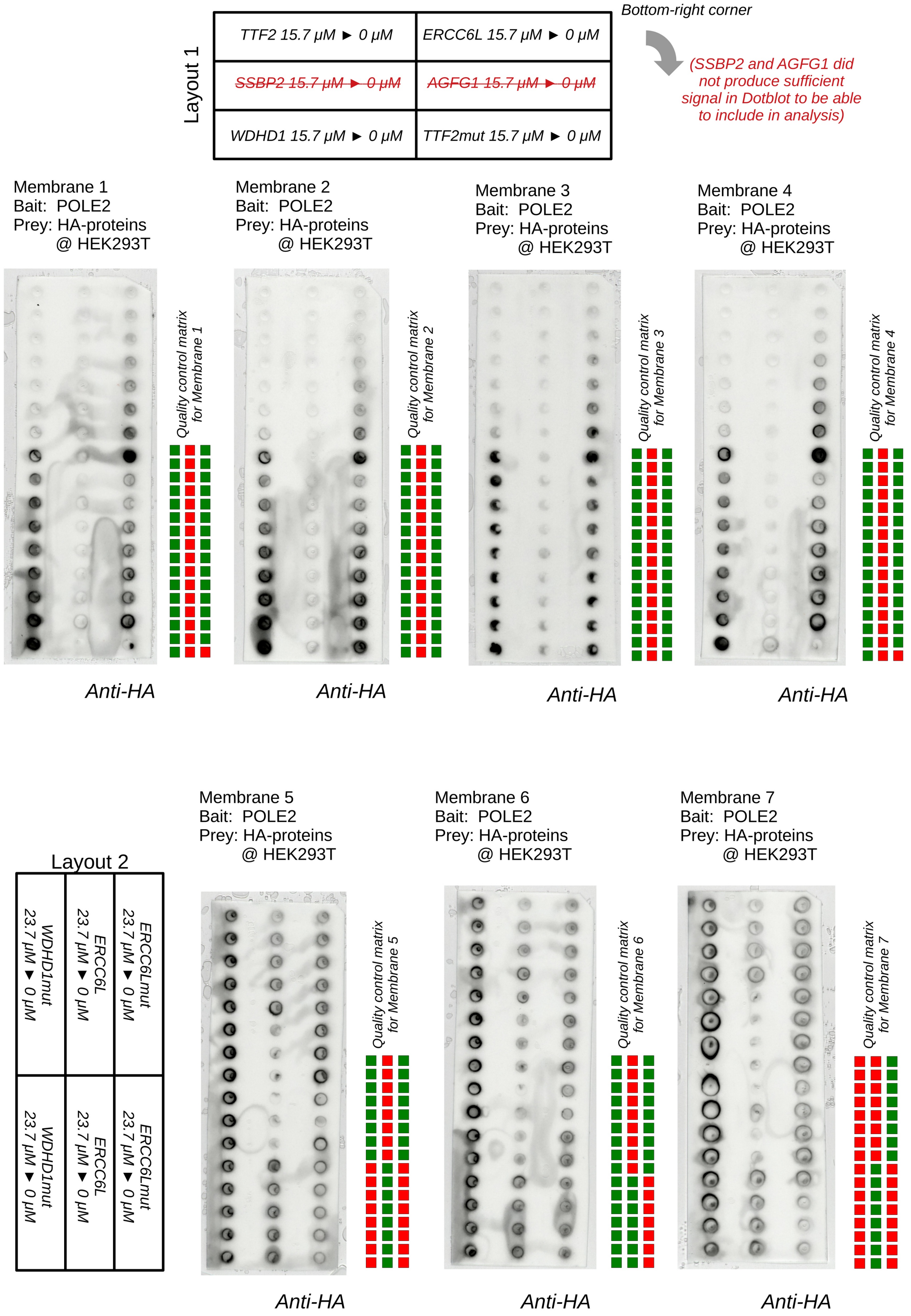

Supplementary Figure 13, Binding of POLE2 to full-length proteins with wild type (NPF) or mutant (KAF) motifs, measured with nHU experiments coupled with dot blot analysis. On a 48 dot screen, 8-point nHU titration experiments of six protein variants are included, however some proteins were not suitable for dot blot quantification. Dot blots were performed in technical triplicates or quadriplicates. Due to technical difficulties, such as because of complete or partial signal loss due to puncture of membrane under vacuum, specific wells were omitted from analysis. This is indicated by a quality control matrix on the right side of each membrane. Source data can be found in the Source Data file.

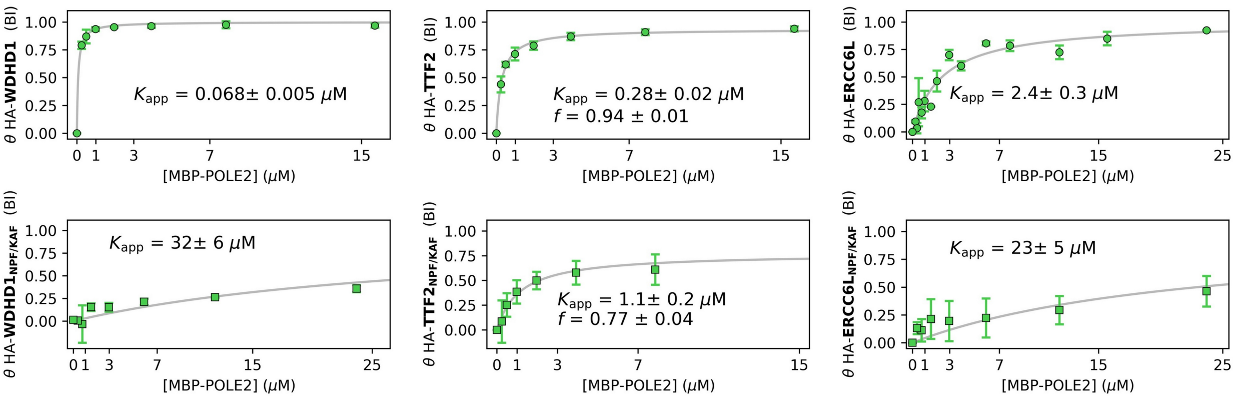

Supplementary Figure 14, Results of nHU titration experiments. Binding isotherms of POLE2 partners. Partial binding activity was used in the case of TTF2 interactions. Source data can be found in Supplementary Data 2, and in the Source Data file.

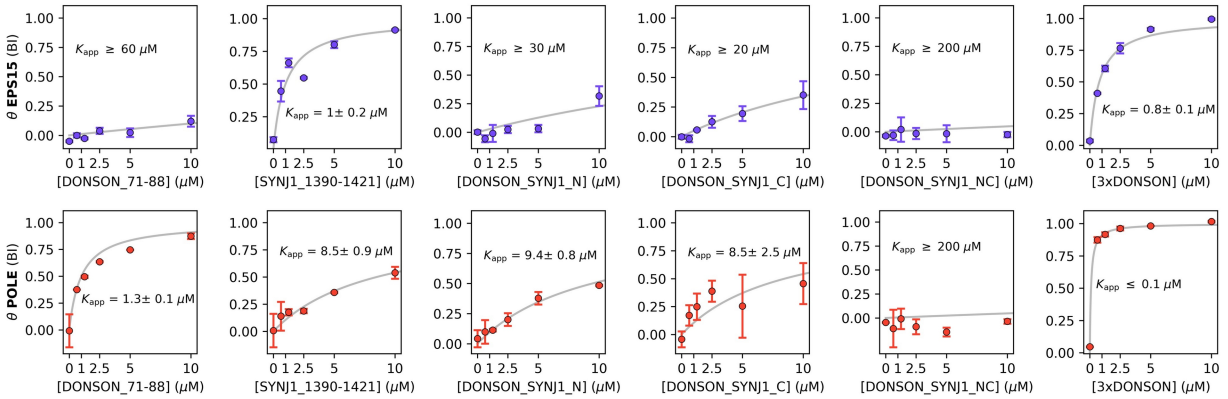

Supplementary Figure 15, Results of nHU titration experiments with SYNJ1 and DONSON chimera NPF motif peptides. The binding of each peptide was measured against EPS15, as well as POLE. Source data can be found in Supplementary Data 2, and in the Source Data file.

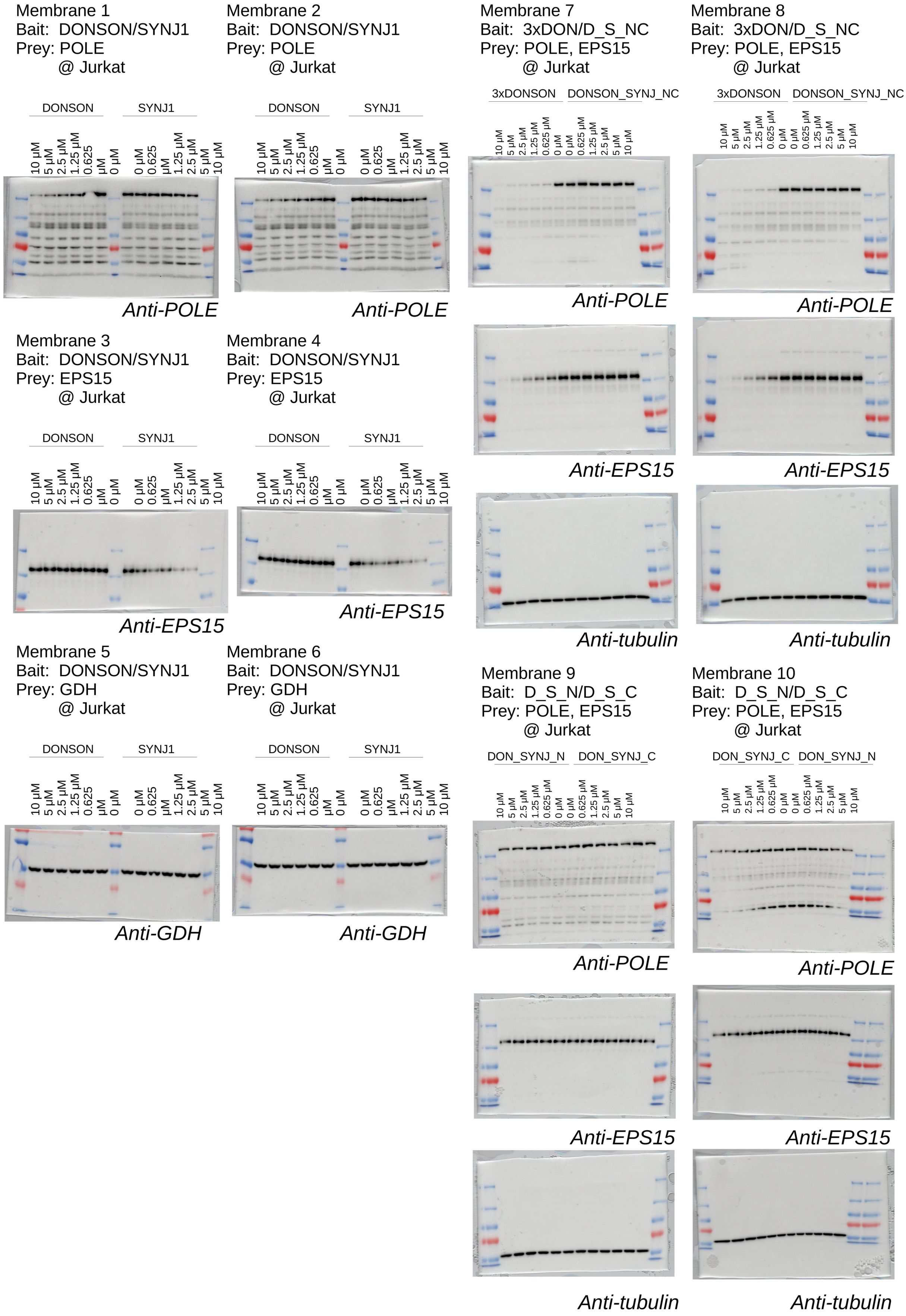

Supplementary Figure 16, Raw data of the nHU-WB experiments shown on Supplementary Figure 15. Western blots were performed in technical duplicates. Western blot images are shown as overlay of luminescence and colorimetric photos. Source data can be found in the Source Data file.

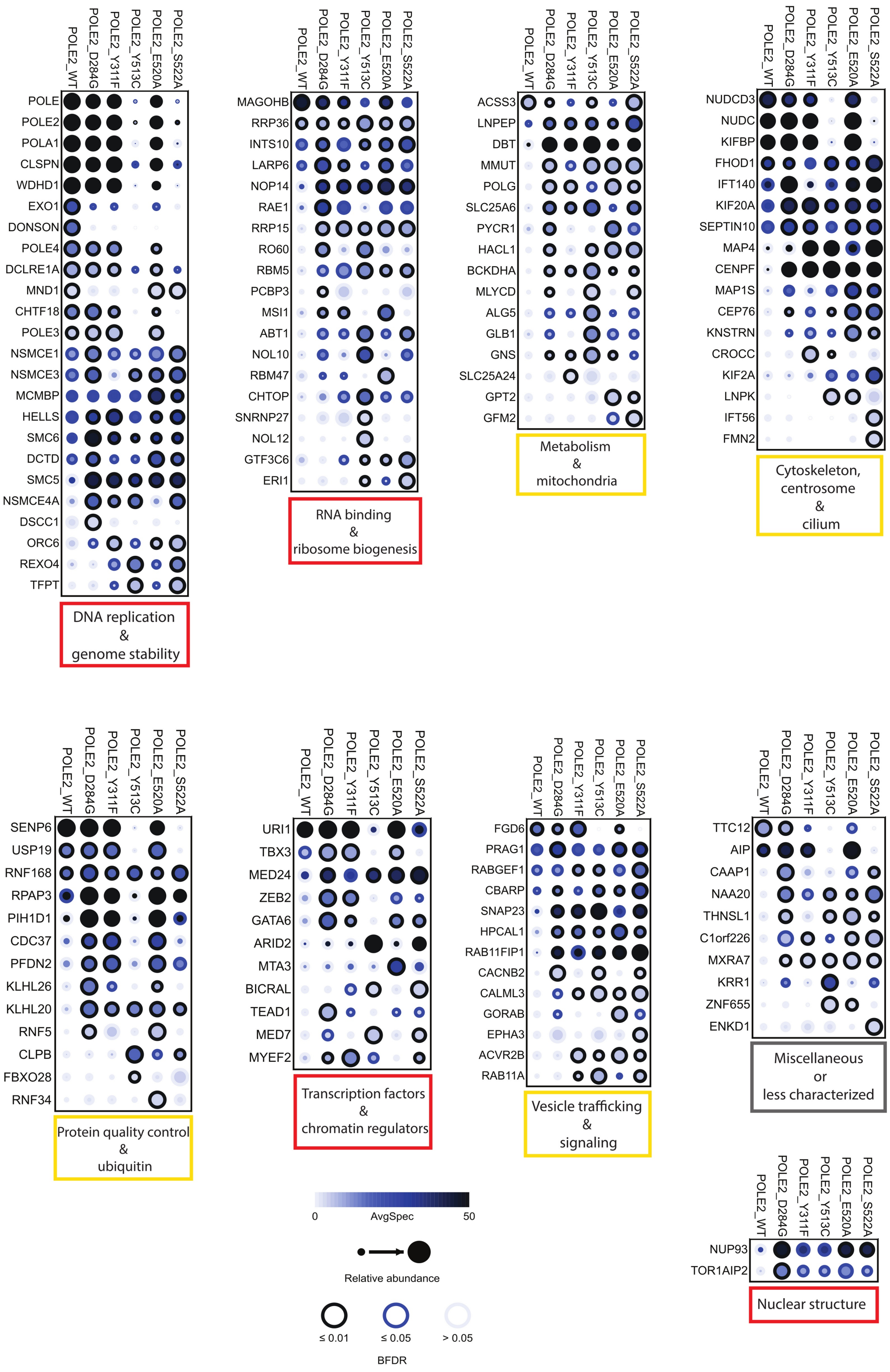

Supplementary Figure 17, Results of proximity labeling experiments reveal extensive POLE2 interactome linked to nuclear and cytoplasmic biological processes. Node size is adjusted based on relative abundance (i.e. measured protein amount), node color based on average spectral count (AvgSpec) values (i.e. measurement reliability) and node edge color based on Bayesian false discovery rate (BFDR) values (i.e. likelihood of false measurement). Protein clustering was done based on Gene Ontology annotations. Selected nuclear and cytoplasmic processes are boxed in red, and yellow, respectively. Proximity labeling experiments were performed with N ≥ 3 for each bait and were compared with a set of 98 composite GFP control samples with or without NLS tag. Source data can be found in Supplementary Data 4, and in the Source Data file.

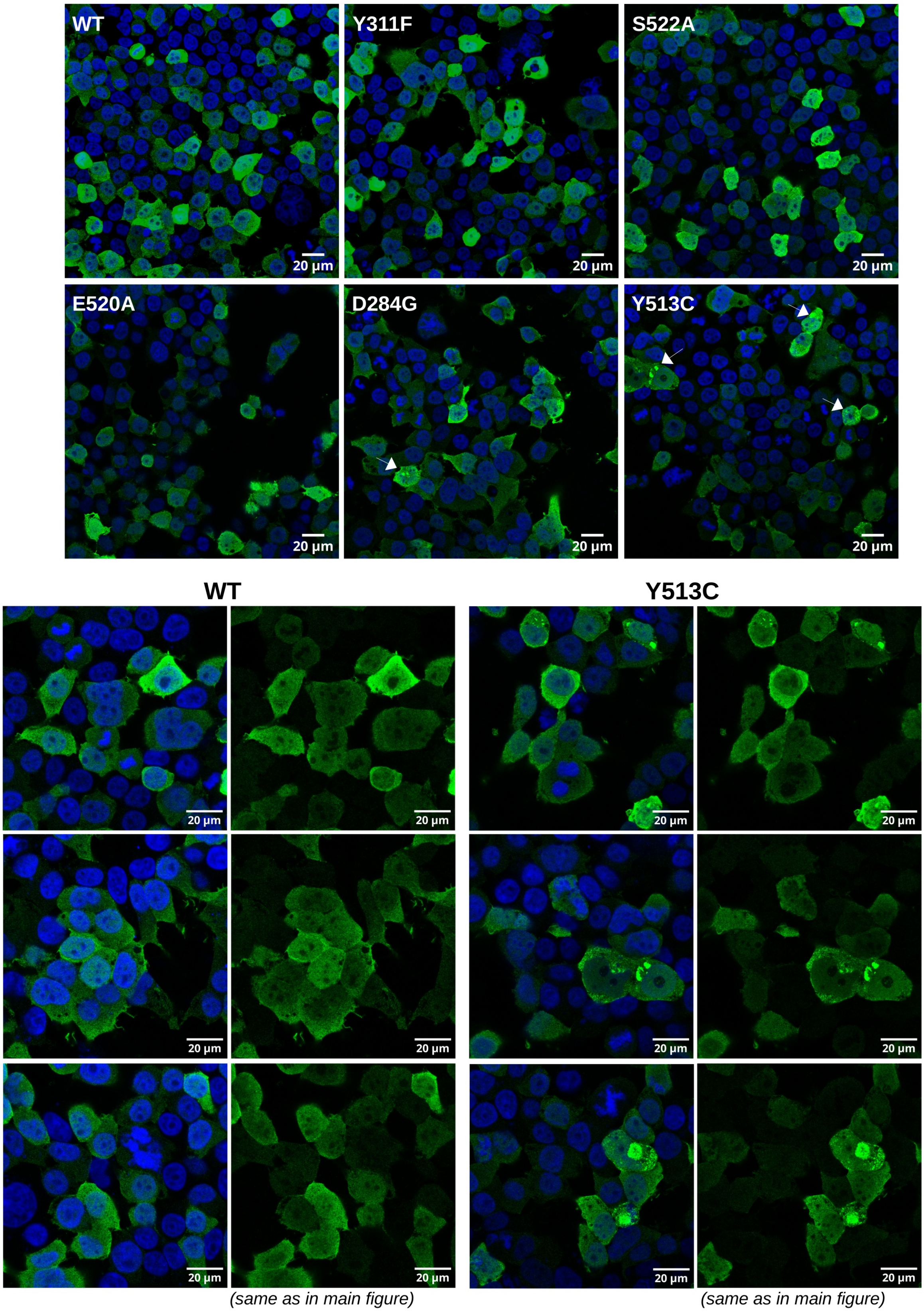

Supplementary Figure 18, Cellular localization of POLE2 variants in HEK293T cells. Transiently transfected cells were stained for HA-POLE2 (in green) and DAPI (in blue) for nucleus. For each variant a confocal image is shown with the overlay of POLE2 and DAPI signals using a low magnification. White arrows indicate observed granular features for Y513C and D284G. On the bottom, zoomed-in views are shown of cells expressing WT and Y513C mutant POLE2 variants with overlay images on the left and only POLE2 signals on the right.

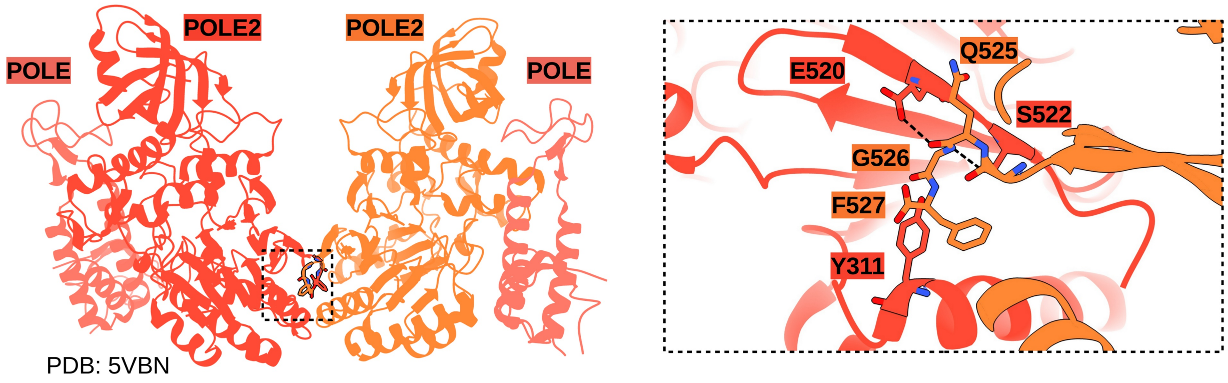

Supplementary Figure 19, POLE2 in crystallo interacts with its own C-terminal tail through the same binding interface used to capture NPF motifs. In a previously solved crystal structure, a POLE2 dimer was observed where the non-conserved C-terminal tail of the protein (sequence: LQGF-COOH) was found to interact symmetrically with the same binding pocket used by NPF motifs, formed by S522, E520, Y311. The last Phe residue critical for such interaction is only present in mammalians and there is no evidence about in solution POLE2 dimers. Thus such interaction seems to be a crystallographic artifact, yet it is likely caused good performance of identifying NPF-mediated interactions in AF3 predictions and it also signifies the capacity of the binding pocket to interact with an aromatic sidechain, such as the one found in NPF or NPY motifs.
