# Supplementary material for: The non-catalytic DNA polymerase ε subunit is an NPF motif recognition protein
Source: Nat Commun. 2025 Dec 13;17:586. doi: 10.1038/s41467-025-67284-5 (PMC12808100; doi:10.1038/s41467-025-67284-5)
Supplement: Supplementary file 2 — Descriptions of Additional Supplementary Files [file 41467_2025_67284_MOESM2_ESM.pdf]

Description of Additional Supplementary Files

Supplementary Data 1 - Results of nHU-MS experiments. Each sheet contains the result of a nHU measurement performed with a specific bait. Significant partners have non-zero values in the pKapp_sign column.

Supplementary Data 2 - Results of nHU-WB and nHU-db experiments. Each sheet contains all measured depletion values of Western or dot blot experiments of a specific figure.

Supplementary Data 3 - Results of the de novo motif discovery approach. The first sheet contains the identified motifs found in disordered sequences of the bait molecules, identified by STREME. The second sheet contains evolutionary scoring of al identified motif instances. The last sheet contains the motif classes re-ranked by their average evolutionary scores.

Supplementary Data 4 - Results of proximity labeling experiments. Results of proximity labeling experiments performed with POLE2 and its variants. High-confidence interactors (HCIs) were defined as proteins with a Bayesian false discovery rate (BFDR) of 0 for each bait. Data for all HCIs across all baits were combined from the SAINT output file and used to generate the dot plots shown in the figures.
